# Die gesellschaftliche Akzeptanz von öffentlichem Stillen im zeitlichen Vergleich: Erfahrungen und Einstellungen der Bevölkerung und stillender Mütter 2016 und 2020

**DOI:** 10.1007/s00103-022-03596-x

**Published:** 2022-10-13

**Authors:** Stephanie Lücke, Severine Koch, Gaby-Fleur Böl, Maria Flothkötter

**Affiliations:** 1 Netzwerk Gesund ins Leben im Bundeszentrum für Ernährung, Deichmanns Aue 29, 53179 Bonn, Deutschland; 2 Abteilung Risikokommunikation, Bundesinstitut für Risikobewertung (BfR), Berlin, Deutschland

**Keywords:** Stillförderung, Akzeptanz, Öffentlichkeit, Wahrnehmung, Trendbefragung, Breastfeeding promotion, Acceptance, Public, Perception, Trend survey

## Abstract

**Hintergrund und Ziel:**

Deutschland ist nur moderat stillfreundlich, dies ergab eine systematische Bestandsaufnahme von 2019. Aufbauend auf einer Referenzstudie wurde eine Befragung zur Akzeptanz des Stillens in der Öffentlichkeit durchgeführt. Ziel der Arbeit ist es, Wahrnehmungen und Einstellungen zum öffentlichen Stillen im zeitlichen Vergleich zu erfassen. Zudem werden u. a. Unterschiede zwischen der Allgemeinbevölkerung und Müttern von kleinen Kindern sowie zwischen Müttern mit unterschiedlichem Bildungsstand betrachtet.

**Material und Methoden:**

1007 Personen ab 16 Jahren und 307 Mütter mit Kindern bis 24 Monaten wurden im Jahr 2020 online zum Thema Stillen in der Öffentlichkeit befragt. Ergebnisse wurden mit einer früheren Befragung aus 2016 verglichen.

**Ergebnisse:**

2020 stillt ein größerer Anteil von Müttern an öffentlichen Orten als 2016, es wird seltener vermieden. Mütter mit niedrigerem Bildungsstand stillen seltener, auch in der Öffentlichkeit, und berichten häufiger von gemischten Reaktionen. In der Bevölkerung ist die Akzeptanz für das öffentliche Stillen gesunken, z. B. in der Gastronomie. Etwa jede bzw. jeder Sechste (17 %) lehnt das öffentliche Stillen explizit ab. Das Wissen über gesundheitliche Effekte des Stillens ist in der Allgemeinbevölkerung niedriger als bei Müttern. Wie 2016 geht Wissen über die positiven Effekte des Stillens mit einer größeren Akzeptanz von öffentlichem Stillen einher.

**Diskussion:**

Die Ergebnisse unterstreichen die Bedeutung von Maßnahmen, etwa um der Bevölkerung Wissen zu vermitteln, das Stillen in Massenmedien und durch positive Vorbilder häufiger zu zeigen und die Lebenswelten von Familien stillfreundlicher zu machen. Bei den Maßnahmen sind Frauen mit niedrigerem Bildungsstand besonders in den Blick zu nehmen.

## Abstract (en)

**Background and objective:**

Germany is only moderately breastfeeding-friendly, according to a systematic inventory from 2019. Based on a reference study, a survey on the acceptance of breastfeeding in public was conducted. The aim of the work is to capture perceptions and attitudes towards public breastfeeding in a periodic comparison. In addition, differences between the general population and mothers of young children as well as between mothers with different levels of education are considered.

**Materials and methods:**

In 2020, 1007 people aged 16 and older and 307 mothers with children up to 24 months were surveyed online about public breastfeeding. The results were compared with a previous survey from 2016.

**Results:**

In 2020, a larger percentage of mothers breastfeed in public than in 2016; it is avoided less often. Mothers with lower levels of education are less likely to breastfeed, also in public, and more likely to report mixed reactions. Among the general population, acceptance of public breastfeeding has declined, for example, in gastronomy. About one in six (17%) explicitly rejects breastfeeding in public. Knowledge about the health effects of breastfeeding is lower among the general public than among mothers. As in 2016, knowledge about the positive effects of breastfeeding is associated with greater acceptance of public breastfeeding.

**Conclusions:**

The results underline the importance of measures, for example, to communicate knowledge to the population, show breastfeeding more frequently in mass media and through positive role models, and make the settings in which families live more breastfeeding-friendly. Measures should pay special attention to women with lower levels of education.

## Hintergrund

Etwa 9 von 10 Müttern in Deutschland legen ihr Kind nach der Geburt zum Stillen an, der natürlichen, von Expertinnen und Experten bevorzugten Ernährungsform für Säuglinge [1, 2]. In den ersten Lebensmonaten sinkt die Zahl der gestillten Säuglinge stark [1, 3]. Als Barriere wird unter anderem das Stillen in der Öffentlichkeit genannt [4–11].

Diese Befunde stützt auch eine Studie aus dem Jahr 2016, in der die Wahrnehmung und Akzeptanz des Stillens in der Öffentlichkeit in der Bevölkerung derer von Müttern von kleinen Kindern gegenübergestellt wurden [12]. Jede zweite Mutter vermied zumindest gelegentlich das Stillen in der Öffentlichkeit. Fast die Hälfte der öffentlich stillenden Mütter berichtete darin von gemischten Reaktionen. Und tatsächlich fand ein messbarer Teil der Bevölkerung – etwa jede bzw. jeder Achte –, dass Stillen in der Öffentlichkeit nichts zu suchen habe.

Im internationalen Forschungsvorhaben Becoming Breastfeeding Friendly (BBF) wurde zwischen 2017 und 2019 eine systematische Bestandsaufnahme zum Stand der Stillförderung in Deutschland vorgenommen [13, 14]. Demnach ist Deutschland bislang nur moderat stillfreundlich, unter anderem weil ein umfassendes Stillmonitoring und Öffentlichkeitsarbeit zur Unterstützung von Stillenden fehlen [15, 16]. Dieses Ergebnis wurde zum Anlass genommen, angelehnt an die o. g. Studie zur Akzeptanz des Stillens in der Öffentlichkeit von 2016 eine neue Befragung durchzuführen.

### Trendbefragung 2016/2020

Die vorliegende Studie wurde vom Bundeszentrum für Ernährung (BZfE) im Vorfeld der Entwicklung einer Kommunikationsstrategie zur Stillförderung initiiert. Sie baut, wie oben erwähnt, auf der im Jahr 2016 durchgeführten Befragung des Bundesinstituts für Risikobewertung (BfR) auf [12] und wurde gemeinsam durchgeführt. Aus dem direkten Vergleich mit der Referenzstudie können Trends zum Stimmungsbild, zu Erfahrungen und Verhaltensmustern in Bezug auf öffentliches Stillen in Deutschland abgeleitet werden.

Die zentralen Fragestellungen der vorliegenden Studie sind, inwiefern sich die Wahrnehmung und Akzeptanz von öffentlichem Stillen an verschiedenen Stillorten, die Einstellungen zum Stillen in der Öffentlichkeit, das Wissen über die gesundheitlichen Effekte des Stillens, Stillpraktiken, Erfahrungen beim öffentlichen Stillen und Vermeidungsverhalten zwischen 2016 und 2020 entwickelt haben und welche Unterschiede dabei zwischen Müttern mit Kindern im Stillalter und der Allgemeinbevölkerung zu erkennen sind. Ergänzend richtet sich die aktuelle Befragung gezielt auf Gruppenvergleiche zwischen Müttern mit unterschiedlichem formalen Bildungsstand, da sich diese Unterscheidung in anderen Untersuchungen als bedeutsam erwiesen hat [4, 17, 18].

## Material und Methoden

### Stichprobe und Durchführung

Die Teilnehmenden wurden aus einem bestehenden Online-Panel rekrutiert und die Befragung wurde mittels computergestützter Online-Interviews durchgeführt. Die Stichprobe im Jahr 2020 gliedert sich wie 2016 in 2 Gruppen: eine Bevölkerungsstichprobe und eine Stichprobe von Müttern mit kleinen Kindern. Aufgrund der Art der Rekrutierung war die Bevölkerungsstichprobe eingeschränkt repräsentativ.

Die Bevölkerungsstichprobe umfasste 1007 deutschsprachige Personen ab 16 Jahren und entsprach im Hinblick auf die Merkmale Geschlecht, Alters- und Bildungsstruktur, Ortsgröße und Region (Nielsengebiete^1^) der Verteilung in der Allgemeinbevölkerung in Deutschland. Der Altersdurchschnitt lag bei 47,6 Jahren (16 bis 75 Jahre) und rund 49 % der Teilnehmenden waren männlich. Rund 58 % der Personen verfügten über einen höheren formalen Bildungsstand (hier definiert als mindestens im Besitz eines Realschulabschlusses mit abgeschlossener Berufsausbildung) und rund 42 % über einen niedrigeren formalen Bildungsstand (hier definiert als höchstens im Besitz eines Realschulabschlusses ohne abgeschlossene Berufsausbildung).

Die zweite Stichprobe setzte sich aus 307 deutschsprachigen Müttern zusammen, deren jüngstes Kind nicht älter als 24 Monate war und die somit zum Zeitpunkt der Befragung oder in den Monaten zuvor gestillt haben könnten. Die Mütter waren zwischen 17 und 43 Jahren alt (*M*_Alter_ = 29,9). Um gezielt Vergleiche zwischen Müttern mit höherem formalen Bildungsstand und Müttern mit niedrigerem formalen Bildungsstand ziehen zu können, waren diese Merkmalsausprägungen je zur Hälfte in der Stichprobe vertreten.

### Fragebogen

Der Fragebogen wurde ähnlich wie in der Referenzstudie 2016 [12] gestaltet. Sowohl die Bevölkerungsstichprobe als auch die Stichprobe der Mütter wurden zur Wahrnehmung und Akzeptanz von öffentlichem Stillen an verschiedenen Stillorten, ihren Einstellungen zum Stillen in der Öffentlichkeit und zum Wissen über die gesundheitlichen Effekte des Stillens befragt. Wie 2016 erhielt die Gruppe der Mütter zudem Fragen zu Stillpraktiken, Erfahrungen beim öffentlichen Stillen und Vermeidungsverhalten.

Ergänzend wurde nach den Gründen für das Vermeiden von öffentlichem Stillen, der Akzeptanz von Aktivitäten zur Stillförderung sowie dem Rauchverhalten gefragt. Wenige Fragen wurden um zusätzliche Antwortkategorien erweitert. So enthielt beispielsweise die Frage zum Wissen über positive Auswirkungen des Stillens weitere Antwortmöglichkeiten zu Effekten auf Mutter und Kind, darunter auch Falschaussagen. Die Falschaussagen wurden ergänzt, um überprüfen zu können, ob eventuelle Gruppenunterschiede sich auf tatsächliche Auswirkungen des Stillens beschränken.

### Datenaufbereitung und Auswertung

Die Datenaufbereitung und Analyse wurden mit der Statistiksoftware SPSS (Version 26, IBM, Armonk, NY, USA) durchgeführt. Für kategorische Variablen wurden statistische Unterschiede anhand von Chi-Quadrat-Tests ermittelt. Für korrelative Analysen wurde Spearmans Rho als Korrelationskoeffizient herangezogen. Als signifikant bezeichnete Effekte haben stets ein Signifikanzniveau von *p* < 0,05, sofern nicht anders angegeben.

## Ergebnisse

### Stillrate 2020 so hoch wie 2016.

Die befragten Mütter mit Kindern bis 24 Monate (*n* = 307) hatten sich mehrheitlich vor der Geburt gewünscht, ihr Kind zu stillen (2020: 88 % (eher) Ja; 2016: 89 % (eher) Ja). Die meisten Mütter (85 % der Befragten) haben diesen Wunsch umsetzen können. Die Stillrate ist somit im Vergleich zu 2016 (87 % Stillende) fast unverändert hoch.

Frauen, die zum Zeitpunkt der Befragung bereits abgestillt hatten (*n* = 182), führten eine nicht ausreichende Milchmenge am häufigsten als Grund für das Abstillen an (56 %; 2016: 62 %). Der am zweithäufigsten genannte Grund war, dass das Kind nicht mehr gestillt werden wollte (32 %; 2020 erstmals abgefragt). Etwa ein Sechstel der Frauen gab an, dass sie selbst nicht mehr stillen wollten (16 %; 2020 erstmals abgefragt). Soziale Gründe wie der Unwille, vor fremden Menschen zu stillen, führten selten zum Abstillen (5 %). Die Bedeutung dieses Beweggrundes hat im Vergleich zu 2016 abgenommen (2016: 10 %). Bei Müttern mit niedrigerem Bildungsstand (*n* = 150) ist die Stillrate mit 78 % signifikant geringer als bei Frauen mit höherem Bildungsstand (93 %; *n* = 157). In der erstgenannten Gruppe ist ein signifikant höherer Anteil an Raucherinnen, dieser Befund deckt sich mit anderen Studien [17, 18].

### Frauen stillen 2020 häufiger an öffentlichen Orten.

Die Frage, ob sie auch unterwegs im Beisein ihnen unbekannter Menschen stillen oder gestillt haben, bejahten 73 % der befragten Mütter mit Stillerfahrung (*n* = 263). Öffentliches Stillen ist damit in der aktuellen Befragung verbreiteter als 2016 (65 %; *p* = 0,051). Vor allem der Anteil *gelegentlich* öffentlich stillender Mütter ist im Vergleich zur vorherigen Befragung angestiegen (2016: 33 %; 2020: 40 %). Mütter mit höherem Bildungsstand stillen 2020 signifikant häufiger öffentlich (80 % zumindest gelegentlich) im Vergleich zu Müttern mit niedrigerem Bildungsstand (65 % zumindest gelegentlich).

### Frauen vermeiden 2020 seltener das Stillen in der Öffentlichkeit.

**Figure 1:**
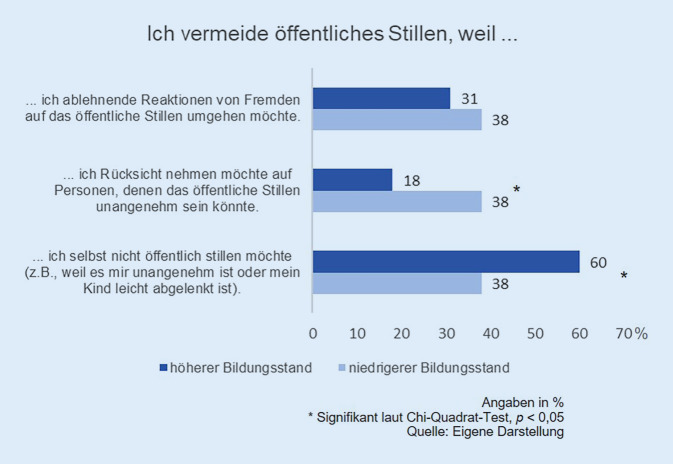

Von den Frauen mit Stillerfahrung an öffentlichen Orten (*n* = 192) gaben 48 % an, das Stillen außer Haus zu vermeiden. Diese Gruppe setzte sich aus Frauen zusammen, die öffentliches Stillen „häufig“ oder „selten“ vermeiden. Auch 2016 mieden knapp die Hälfte der Mütter das öffentliche Stillen (46 %). Jedoch ist seit 2016 der Anteil derer signifikant gestiegen, die das Stillen in der Öffentlichkeit nur „selten“ vermeiden (2016: 24 %; 2020: 33 %). Hingegen gaben weniger Mütter an, das Stillen außer Haus „häufig“ zu vermeiden (2016: 22 %; 2020: 15 %; *p* = 0,071). Während Mütter mit höherem Bildungsstand das Stillen außer Haus signifikant häufiger aus eigener Entscheidung umgehen, möchten Mütter mit niedrigerem Bildungsstand signifikant häufiger soziale Reaktionen vermeiden – also mit ihrem Verhalten vorsorglich auf andere Menschen Rücksicht nehmen (Abb. 1).

### Mütter stillen 2020 häufiger in Geschäften, meiden weiterhin den Nahverkehr.

**Figure 2:**
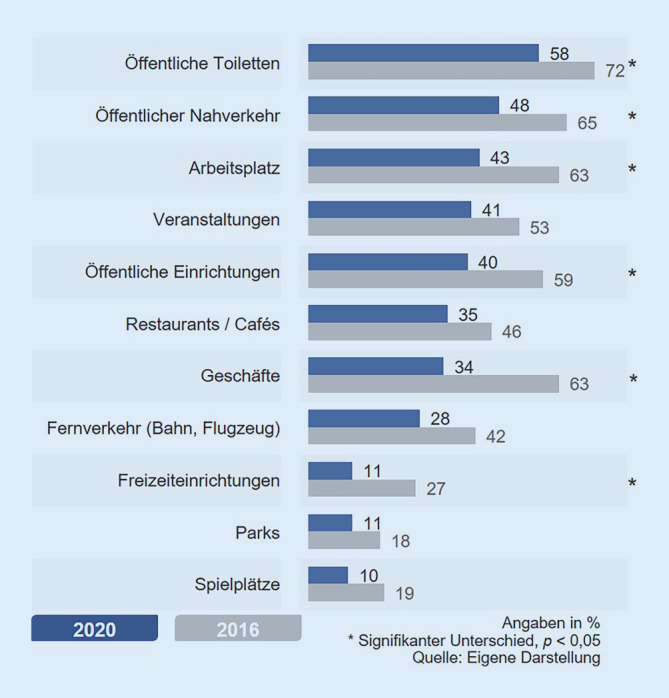

Mütter, die angaben, das Stillen in der Öffentlichkeit zu meiden bzw. gemieden zu haben, wurden gefragt, an welchen Orten sie besonders ungern stillen. So vermeiden 58 % das Stillen auf öffentlichen Toiletten, 48 % im öffentlichen Nahverkehr und 43 % am Arbeitsplatz (Abb. 2). Im Vergleich zum Antwortverhalten 2016 zeigt sich jedoch, dass das Stillen an allen öffentlichen Orten seltener vermieden wird. Vor allem Geschäfte, öffentliche Einrichtungen und der Arbeitsplatz werden signifikant seltener gemieden als 4 Jahre zuvor (19 bis 29 Prozentpunkte Unterschied).

### Viele Frauen erleben gemischte Reaktionen beim öffentlichen Stillen.

Überwiegend negative Reaktionen beim Stillen in der Öffentlichkeit erlebt ähnlich wie 2016 nur ein geringer Teil der Frauen (2016: 6 %; 2020: 5 %). Gemischte Erfahrungen sind dahingegen sehr verbreitet: 40 % der Frauen berichteten von teils positiven und teils negativen Reaktionen beim Stillen in der Öffentlichkeit (2016: 37 %). Unter ihnen sind im relativen Vergleich signifikant mehr Mütter mit niedrigerem Bildungsstand (50 %; *n* = 38) als Mütter mit höherem Bildungsstand (33 %, *n* = 38).

Alle Frauen, die zumindest teils negative Erfahrungen beim öffentlichen Stillen gemacht haben (*n* = 147), sollten diese Erfahrungen genauer beschreiben. Knapp 70 % berichteten dabei von missbilligenden Blicken, während sich gut ein Viertel kritischen Bemerkungen von anderen Menschen ausgesetzt sah. Etwa 10 % der Befragten haben Beschimpfungen, eine gleichgültige Atmosphäre oder die Bitte um einen Ortswechsel erlebt. Insgesamt glichen die Ergebnisse denen von 2016.

### Die Bevölkerung nimmt öffentlich Stillende deutlich seltener wahr.

**Figure 3:**
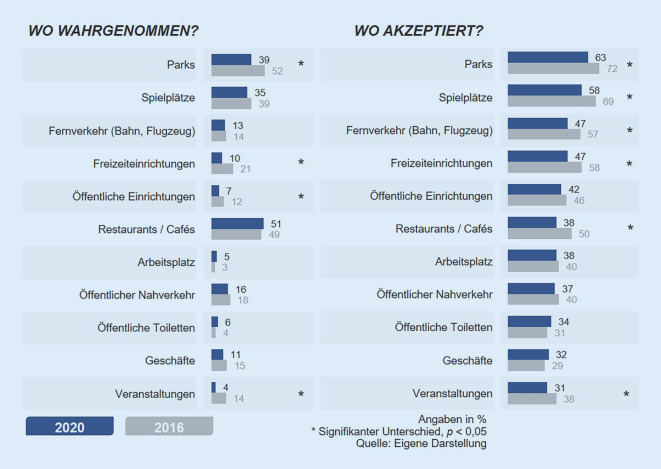

Im Gegensatz zum gestiegenen Anteil öffentlich stillender Mütter werden diese jedoch im Jahr 2020 von der Bevölkerung kaum wahrgenommen: Nur 28 % der Bevölkerungsstichprobe geben an, in den letzten 12 Monaten eine ihnen unbekannte Frau gesehen zu haben, die ein Kind stillt. Im Jahr 2016 hatten noch 51 % der Befragten mindestens eine stillende Frau in der Öffentlichkeit wahrgenommen, also fast doppelt so viele. Vor allem an solchen Orten sinkt die Wahrnehmung von Stillenden signifikant, die während der Corona-Pandemie seltener aufgesucht wurden oder zeitweise geschlossen waren: in Freizeiteinrichtungen wie Schwimmbädern, öffentlichen Einrichtungen wie Bürgerämtern oder bei Veranstaltungen. Nur in Parks, auf Spielplätzen und in der Gastronomie werden stillende Frauen überhaupt in merklichem Ausmaß wahrgenommen (35 bis 51 % der Bevölkerungsstichprobe; Abb. 3).

### Auch die Akzeptanz des öffentlichen Stillens in der Bevölkerung sinkt.

Nicht nur die Wahrnehmung von Stillenden in der Öffentlichkeit ist gesunken, sondern ebenso die Akzeptanz seitens der Bevölkerung. An fast allen abgefragten Orten sprechen sich im Jahr 2020 weniger Befragte dafür aus, dass Frauen dort stillen sollten (Abb. 3). Im Vergleich zu 2016 sinkt die Akzeptanz signifikant sowohl für das Stillen draußen (z. B. Spielplätze) als auch drinnen (etwa Cafés/Restaurants). Das Vermeidungsverhalten Stillender und die Akzeptanz durch andere haben sich somit *gegenläufig* entwickelt.

### Zwei Drittel für öffentliches Stillen, jede(r) Sechste generell dagegen.

**Figure 4:**
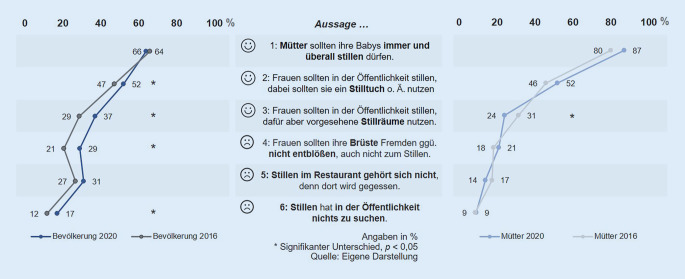

Von der Bevölkerungsstichprobe (*n* = 1007) stimmen etwa zwei Drittel explizit der Aussage zu, dass Mütter ihre Babys immer und überall stillen dürfen (64 %: stimme voll und ganz/eher zu bei Aussage 1; Abb. 4). In der Mütterstichprobe (*n* = 307) zeigt sich eine signifikant stärkere Zustimmung (87 %: stimme voll und ganz/eher zu; Aussage 1; Signifikanz in Abb. 4 nicht dargestellt). Jede bzw. jeder sechste Befragte stimmt explizit der Aussage zu, dass Stillen in der Öffentlichkeit nichts zu suchen hat (17 %: stimme voll und ganz/eher zu; Aussage 6). Das ist ein signifikanter Anstieg im Vergleich zum Jahr 2016 (12 % Zustimmung). In der Mütterstichprobe liegt die Zustimmung zu beiden Befragungszeitpunkten niedriger, bei jeweils 9 %.

### Bedingungen für öffentliches Stillen steigen und werden nur teilweise von Müttern geteilt.

Etwa ein Drittel der Bevölkerung knüpft Bedingungen an das Stillen in der Öffentlichkeit: dafür *Stillräume* zu nutzen, die Brüste *nicht zu entblößen* oder *Restaurants* dafür zu *meiden* (29–37 %: stimme voll und ganz/eher zu; Aussagen 3 bis 5). Hier zeigt sich wie 2016 das Konfliktpotenzial in der Gastronomie, denn mit 14 % stimmen signifikant weniger Mütter der Aussage 5 zu: „Stillen im Restaurant gehört sich nicht, denn dort wird gegessen“ (Bevölkerung: 31 %; Signifikanz in Abb. 4 nicht dargestellt). Hinsichtlich der Nutzung von *Stilltüchern* sind sich hingegen Mütter mit der Bevölkerung einig: Jeweils 52 % sprechen sich für das öffentliche Stillen aus, wenn Stilltücher verwendet werden (Aussage 2).

Allen genannten Bedingungen an das Stillen schließen sich im Jahr 2020 mehr Menschen an als 2016 (Zunahme der Zustimmung um 4 bis 9 Prozentpunkte, Aussagen 2 bis 5 ggü. 2016; Differenzen bei Aussagen 2 bis 4 signifikant; Abb. 4). In der Mütterstichprobe ist kein einheitlicher Trend zu beobachten: Einerseits ist bei ihnen signifikant die Zustimmung gesunken, sich dafür in *Stillräume* zurückzuziehen (um 7 Prozentpunkte im Vergleich zu 2016; Aussage 3); andererseits ist die Forderung nach der Nutzung von *Stilltüchern* gestiegen (um 6 Prozentpunkte; Aussage 2; *p* = 0,098). Diese Einstellung spiegelt sich auch im Verhalten der befragten Mütter mit öffentlicher Stillerfahrung (*n* = 192): Während 2016 noch 71 % von ihnen häufig oder gelegentlich Stilltücher nutzten, verwenden inzwischen 82 % dieses Hilfsmittel (signifikanter Anstieg um 11 Prozentpunkte).

### Große Mehrheit für Kennzeichnung stillfreundlicher Orte.

2020 wurde erstmals nach der Einstellung zur Kennzeichnung stillfreundlicher Orte gefragt. Mehr als 3 Viertel der Bevölkerungsstichprobe finden Aufkleber zur Kennzeichnung „sehr gut“ (47 %) oder „eher gut“ (32 %). Unter den befragten Müttern ist die Zustimmung mit rund 90 % noch signifikant höher (72 % „sehr gut“; 18 % „eher gut“). Letztere begrüßen auch die Idee einer App, die besonders stillfreundliche Orte und Stillräume in der Umgebung anzeigt: 59 % finden die Idee „sehr gut“, weitere 26 % „eher gut“.

Bei Müttern mit niedrigerem Bildungsstand findet eine Kennzeichnung stillfreundlicher Orte signifikant mehr Zuspruch: 80 % finden es „sehr gut“ (*n* = 120), im Vergleich zu 64 % der Mütter mit höherem Bildungsstand (*n* = 100). Ebenfalls signifikant stärker ist die Zustimmung der Mütter mit niedrigerem Bildungsstand zu einer App, die stillfreundliche Orte und Stillräume anzeigt: 69 % finden die Idee „sehr gut“ (*n* = 103), hingegen nur 50 % der Mütter mit höherem Bildungsstand (*n* = 79).

### Wissen über positive gesundheitliche Effekte des Stillens auf Kind und Mutter teils lückenhaft.

**Figure 5:**
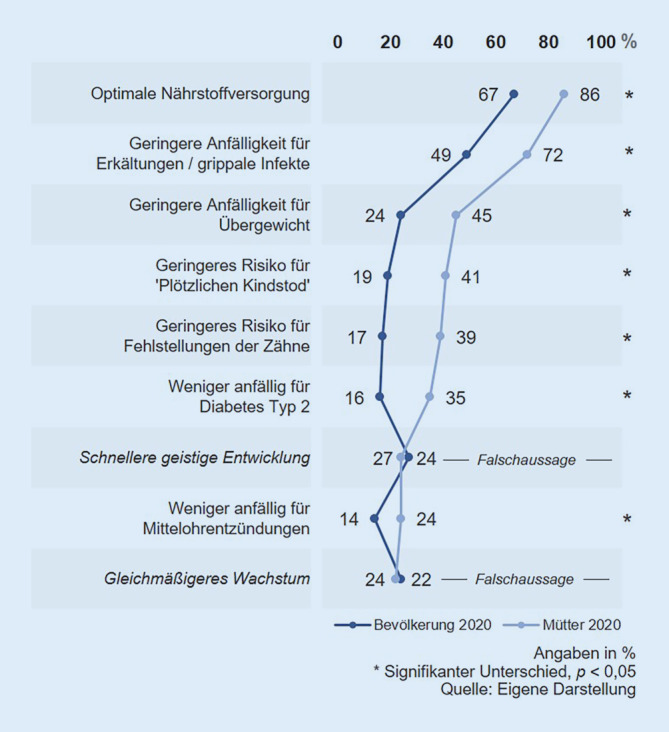

Es wurde die Bekanntheit von 9 möglichen gesundheitlichen Auswirkungen des Stillens auf das Kind abgefragt, darunter 2 falsche (Abb. 5). Alle tatsächlichen Effekte auf das Kind sind den Müttern signifikant häufiger bekannt als der Bevölkerungsstichprobe, während die 2 falschen Auswirkungen von beiden Gruppen etwa gleich häufig für wahr gehalten wurden (22–27 % Zustimmung).

Das Wissen um eine optimale Nährstoffversorgung des Kindes durch das Stillen ist in der Bevölkerungsstichprobe im Vergleich zur vorherigen Befragung signifikant gesunken (2016: 82 % Bekanntheit; 2020: 67 % Bekanntheit). Unter Müttern ist das Wissen um diese positive Auswirkung etwa gleich hoch geblieben (2016: 89 %; 2020: 86 %).

Fast alle abgefragten positiven Effekte des Stillens auf das Kind sind Müttern mit höherem Bildungsstand signifikant häufiger bekannt als Müttern mit niedrigerem Bildungsstand, darunter auch ein falscher. Das Wissen um eine optimale Nährstoffversorgung des Kindes durch das Stillen war in beiden Gruppen weitverbreitet (niedrigerer Bildungsstand: 87 %; höherer Bildungsstand: 86 %). Größere Wissensunterschiede sind insbesondere bei den verringerten Risiken des Kindes für den plötzlichen Kindstod, Übergewicht und Diabetes Typ 2 zu erkennen.

**Figure 6:**
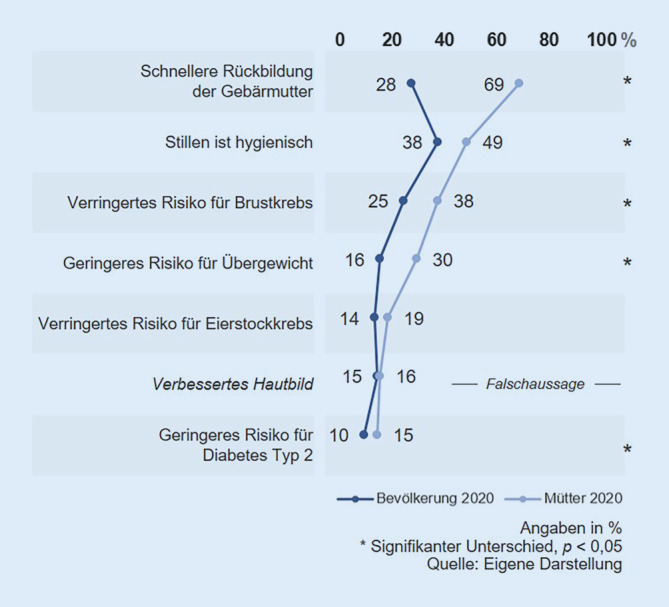

In Bezug auf die gesundheitlichen Auswirkungen des Stillens auf die Mutter wurde die Bekanntheit von 8 möglichen Auswirkungen abgefragt, darunter eine falsche (Abb. 6). Den Müttern sind fast alle tatsächlichen Effekte auf die Mutter signifikant häufiger bekannt als der Bevölkerungsstichprobe.

Den größten Wissensvorsprung haben Mütter hinsichtlich der Rückbildung der Gebärmutter. Diese positive Auswirkung ist 69 % der Mütter, aber lediglich 28 % der Bevölkerungsstichprobe bekannt. Auch die verringerten Risiken für Brustkrebs und Übergewicht sind den Müttern deutlich häufiger bekannt als der Bevölkerungsstichprobe.

Insgesamt fällt auf, dass das Wissen über die gesundheitlichen Auswirkungen auf die Mutter eher gering ausfällt, nicht nur in der Bevölkerungsstichprobe, sondern auch bei den befragten Müttern. Abgesehen von der schnelleren Gebärmutterrückbildung sind sämtliche Effekte weniger als der Hälfte der befragten Mütter bekannt.

Alle abgefragten tatsächlichen Effekte des Stillens auf die Mutter sind Müttern mit höherem Bildungsstand signifikant häufiger bekannt als Müttern mit niedrigerem Bildungsstand. Größere Wissensunterschiede waren insbesondere bei den verringerten Risiken für Brust- und Eierstockkrebs zu erkennen.

### Wissen über Effekte hängt mit Akzeptanz von öffentlichem Stillen zusam‑men.

Mehr Wissen über die positiven gesundheitlichen Effekte des Stillens geht mit einer größeren Akzeptanz von öffentlichem Stillen einher. So erhielt die Aussage: „Mütter sollten ihre Babys immer und überall stillen dürfen“, in der Bevölkerungsstichprobe Zustimmung von 75 % der Personen, denen viele (d. h. mindestens 4) dieser Auswirkungen des Stillens bekannt sind (*n* = 393). Bei Personen, denen nur wenige (1–3; *n* = 451) oder keine dieser Auswirkungen des Stillens bekannt sind (*n* = 163), lag die Zustimmung dahingegen signifikant niedriger bei 59 % bzw. 49 %. Anders ausgedrückt, je mehr Auswirkungen des Stillens den Befragten bekannt waren, desto positiver war auch ihre Einstellung zum öffentlichen Stillen (Spearman-Korrelation = 0,26, *p* < 0,001).

Auch die Akzeptanz für öffentliches Stillen an konkreten Orten hängt mit dem Wissen über die gesundheitlichen Effekte des Stillens zusammen (Spearman-Korrelation = 0,23, *p* < 0,001). So wird das Stillen in Restaurants von rund 54 % der Personen akzeptiert, denen mindestens 4 der positiven gesundheitlichen Auswirkungen des Stillens bekannt sind. Unter den Personen, denen nur wenige oder keine dieser Auswirkungen des Stillens bekannt sind, ist die Akzeptanz für das Stillen in Restaurants mit 38 % bzw. 32 % signifikant niedriger.

## Diskussion

### Mehr öffentliches Stillen, aber weniger Akzeptanz: Stillfreundlichkeit stärker fördern.

Die Daten der vorliegenden Trendbefragung zeigen, dass Mütter im Jahr 2020 in Deutschland häufiger in der Öffentlichkeit stillen als 2016. Sie vermeiden das öffentliche Stillen seltener und nur noch für jede zwanzigste Befragte ist es ein Grund zum Abstillen – 2016 war es noch jede zehnte. Diese neuen Erkenntnisse deuten darauf hin, dass das Stillen in der Öffentlichkeit für die 2020 befragten Mütter selbstverständlicher geworden ist. Gleichzeitig berichten wie 2016 fast die Hälfte von ihnen über gemischte und damit auch negative Reaktionen in ihrem sozialen Umfeld.

In der Bevölkerung werden Stillende 2020 im Vergleich zu 2016 deutlich seltener wahrgenommen. Das bedeutet jedoch nicht, dass es nun selbstverständlich „dazugehört“. Denn gleichzeitig ist auch die gesellschaftliche Akzeptanz für das Stillen sowohl draußen als auch in geschlossenen Räumen gesunken. Das 2016 festgestellte Konfliktpotenzial etwa in der Gastronomie oder in Geschäften hat sich 2020 verschärft (Abb. 2 und 3). Zudem wird das Stillen stärker an Bedingungen geknüpft, etwa ein Stilltuch zu nutzen oder Restaurants zu meiden. Jede bzw. jeder sechste Befragte und damit ein relevanter Teil der Bevölkerung lehnt das Stillen in der Öffentlichkeit generell ab. Der Vergleich zwischen 2016 und 2020 ermöglicht auch einen ersten Einblick in die veränderte Öffentlichkeit in Deutschland durch das Einsetzen der Corona-Pandemie.

Wie in der Erhebung von 2016 weisen auch im Jahr 2020 Befragte mit einer explizit befürwortenden Haltung zum Stillen in der Öffentlichkeit *keine* signifikanten demografischen Unterschiede auf im Vergleich zu Befragten mit einer explizit ablehnenden Haltung.^2^ Somit lässt sich keine spezifische Zielgruppe für eine gezielte Ansprache identifizieren.

Diese Entwicklungen und Befunde erschweren es, bestehende Barrieren für Familien abzubauen und eine stillfreundlichere gesellschaftliche Atmosphäre zu erreichen. Um einen aus Sicht von Familien, Politik und Medizin wünschenswerten Einstellungswandel hin zu einem verständnisvolleren Klima zu fördern, erscheinen Maßnahmen auf mehreren Ebenen sinnvoll [19, 20]:

- *kognitiv*, indem Stillwissen vermittelt wird,
- *affektiv*, indem Stillen mit positiven Emotionen verknüpft wird,
- *konativ*, indem stillfreundliches Verhalten vorgelebt und erleichtert wird.

### *Kognitiv:*

*Wissen über positive Effekte und Stillpraxis vermitteln.* Wie auch 2016 zeigen die Daten: Wer mehr über die gesundheitlichen Effekte weiß, steht dem Stillen im öffentlichen Raum positiver gegenüber. Der erneute Befund legt daher nahe, in der Allgemeinbevölkerung gezielt Wissen zu verbreiten über die positiven Auswirkungen des Stillens auf Kinder und auf Mütter, etwa um dem signifikant gesunkenen Wissen über die optimale Nährstoffversorgung entgegenzuwirken. Auch mehr Wissen über Stillpraktiken kann eine stillfreundliche Atmosphäre begünstigen, z. B. dass manche Säuglinge keine Tücher als Bedeckung beim Stillen akzeptieren. Eine entscheidende Rolle spielen Fachkräfte, die (werdende) Familien begleiten: Auch sie sollen über die Bedeutung und Praxis des Stillens gut informiert sein, um Mütter in ihrem Handeln stärken zu können.

### *Affektiv:*

*Das Stillen selbstverständlich zeigen.* Neben der kognitiven Ebene bieten sich auch Maßnahmen an, die menschliche Emotionen ansprechen [21], etwa im Fernsehen beiläufig positive Fallbeispiele von öffentlich Stillenden in Handlungsstränge einzubetten [22]. Fiktive Geschichten ermöglichen Außenstehenden, die Perspektive zu wechseln und Empathie mit Müttern aufzubauen. Eine solche positive Rahmung („Framing“; [23]) prägt unbewusst die Wahrnehmung dessen, was Menschen als normal empfinden („Kultivierung“; [24]). Fallbeispiele erscheinen insbesondere für Personen relevant, die keinen eigenen Bezug zum Thema Stillen haben [22, 25]. Auch bekannte Persönlichkeiten aus Politik, Sport und Gesellschaft können mit persönlichen Erfahrungsberichten die Akzeptanz für Stillende in der Gesellschaft fördern [26].

### *Konativ:*

*Lebenswelten stillfreundlicher machen.* Stillfördernde Maßnahmen setzen idealerweise dort an, wo sich Stillende häufig bewegen, etwa in kommunalen Settings, am Arbeitsplatz, in Cafés oder Kindertagesstätten. Die in diesen Lebenswelten tätigen Personen können für die Bedeutung des Stillens und die Bedürfnisse von Müttern mit Kindern im Stillalter sensibilisiert werden. Entsprechende Orte können durch Stillecken oder -räume ergänzt oder besonders stillfreundliche Orte symbolisch gekennzeichnet werden. Eine kommunikative Herausforderung ist es, dabei deutlich zu machen, dass sich das Stillen nicht auf diese Orte beschränken soll, man sich dort jedoch besonders für Mütter engagiert.

### Unterschiede im Stillverhalten nach Bildungsstand abbauen.

Mütter mit niedrigerem Bildungsstand stillen insgesamt seltener, kürzer und geben ihrem Kind seltener in der Öffentlichkeit die Brust als ihre Vergleichsgruppe mit höherem Bildungsstand (siehe auch [4, 17, 18]). Zudem *wissen* diese Mütter weniger über die gesundheitlichen Effekte des Stillens als höher gebildete Mütter. Auch eher auf *affektiver* Ebene zu verortende Differenzen zeigen sich: Frauen mit niedrigerem Bildungsstand geben häufiger an, gemischte Reaktionen beim öffentlichen Stillen erlebt zu haben, und vermeiden das Stillen eher, um soziale Reaktionen Dritter zu vermeiden. In dieser Gruppe ist das Interesse an der Kennzeichnung stillfreundlicher Orte messbar stärker als bei Müttern mit höherem Bildungsstand. Einer aktuellen qualitativen Studie zufolge nehmen Frauen mit geringerem Sozialstatus (definiert anhand niedrigen Bildungsstands und Zuordnung als einkommensarm [4]) sozialen Druck von außen ebenso wahr wie Frauen mit höherem Sozialstatus, Letztere weisen diesen Druck jedoch selbstbewusster von sich [4]. Möglicherweise reagieren Stillende mit niedrigerem Bildungsstand sensibler auf Reaktionen der sozialen Umwelt als die Vergleichsgruppe. Für Frauen mit niedrigerem Bildungsstand sind also sämtliche oben beschriebenen Aktivitäten in besonderem Maß bedeutend.

### Nationale Strategie zur Stillförderung berücksichtigt vorgeschlagene Maßnahmen.

Im Sommer 2021 wurde durch die Bundesregierung die „Nationale Strategie zur Stillförderung“ verabschiedet [27]. Im Einklang mit den Ergebnissen dieser Studie umfasst diese auch oben genannte Maßnahmen und nimmt Mütter mit niedrigerem Bildungsstand besonders in den Blick.

### Besonderheiten durch die Corona-Pandemie.

Zum Zeitpunkt der Befragung im September 2020 waren seit etwa 6 Monaten pandemiebedingte Veränderungen zu beobachten, etwa ausgedehnte Home-Office-Regelungen und reduzierte Mobilität [28, 29]. Der festgestellte massive Rückgang der Wahrnehmung von Stillenden im öffentlichen Raum durch die Bevölkerung hängt möglicherweise auch damit zusammen, dass viele Menschen weniger Gelegenheit hatten, Stillenden im öffentlichen Raum zu begegnen, auch wenn 2020 mehr Frauen als 2016 angaben, öffentlich zu stillen. Auch die gesunkene Akzeptanz des öffentlichen Stillens könnte eine Folge der Pandemie sein, etwa weil aufgrund höherer Hygiene-Ansprüche die Gabe einer Körperflüssigkeit wie Muttermilch kritischer gesehen wird.

### Methodische Einschränkungen

Die Befragung wurde mithilfe eines Online-Panels realisiert und ist damit gemäß wissenschaftlicher Gütekriterien nicht repräsentativ für die Grundgesamtheit der Bevölkerung in Deutschland. Immerhin sind jedoch inzwischen 94 % der Bevölkerung online erreichbar [30]. Zudem kann aufgrund der Größe des gewählten Panels (200.000 Personen) und der umfangreichen soziodemografischen Quotierung der Stichprobe darauf geschlossen werden, dass die Ergebnisse einer Repräsentativität nahekommen. Eine weitere Einschränkung der gewählten Methode ist, dass Befragungen im Gegensatz zu Experimenten nur Zusammenhänge zeigen, aber keine kausalen Rückschlüsse zulassen.

## Fazit

Die Befunde der vorliegenden Trendbefragung von 2016 und 2020 unterstreichen die Notwendigkeit, Basiswissen über Stillpraktiken und positive Effekte des Stillens zu vermitteln, da Wissen mit höherer Akzeptanz einhergeht. Darüber hinaus sind Anstrengungen zur Erhöhung der Akzeptanz des Stillens in der Öffentlichkeit zu unternehmen, um eine stillfreundlichere Atmosphäre zu fördern. Zudem sollen Maßnahmen zur gezielten Unterstützung von Frauen mit niedrigerem Bildungsstand ergriffen werden. Eine weitere Erhebungswelle könnte mögliche Effekte in einigen Jahren nach der Umsetzung von Maßnahmen der Nationalen Strategie zur Stillförderung prüfen.

## Footnotes

1 Die Nielsengebiete sind eine regionale Aufteilung nach Verbraucherverhalten und umfassen in Deutschland jeweils ein oder mehrere Bundesländer.

2 Vergleichsgruppen gemessen etwa an Aussage 1: „Mütter sollten ihre Babys immer und überall stillen dürfen“, Abb. 4.
